# Supplementary material for: Assessing the hospital surge capacity of the Kenyan health system in the face of the COVID-19 pandemic
Source: PLoS One. 2020 Jul 20;15(7):e0236308. doi: 10.1371/journal.pone.0236308 (PMC7371160; doi:10.1371/journal.pone.0236308)
Supplement: S1 Table — (DOCX) [file pone.0236308.s001.docx]

**S1 Table: Data on Hospital, ICU beds, and ventilators in Kenya**

|  | **General hospital beds*** | | | | **ICU Beds**** | | | | **Ventilators**** | | | |
| --- | --- | --- | --- | --- | --- | --- | --- | --- | --- | --- | --- | --- |
| **County** | **Public facilities** | **For profit facilities** | **Faith based facilities** | **Total** | **Public facilities** | **For profit facilities** | **Faith based facilities** | **Total** | **Public facilities** | **For profit facilities** | **Faith based facilities** | **Total** |
| Baringo | 284 | 9 | 64 | 357 | 0 |  |  | 0 |  |  |  | 0 |
| Bomet | 229 | 6 | 200 | 435 | 0 |  |  | 0 |  |  |  | 0 |
| Bungoma | 287 | 389 | 130 | 806 | 0 | 24 |  | 24 |  | 8 |  | 8 |
| Busia | 460 | 14 | 96 | 570 | 0 |  |  | 0 |  |  |  | 0 |
| Elgeyo-Marakwet | 233 | 82 | 126 | 441 | 0 |  |  | 0 |  |  |  | 0 |
| Embu | 587 | 23 | 158 | 768 | 5 |  |  | 5 |  |  |  | 0 |
| Garissa | 291 | 4 | 98 | 393 | 6 |  |  | 6 |  |  |  | 0 |
| Homa Bay | 490 | 30 | 160 | 680 | 0 |  |  | 0 |  |  |  | 0 |
| Isiolo | 297 | 14 | 45 | 356 | 0 |  |  | 0 |  |  |  | 0 |
| Kajiado | 268 | 112 | 25 | 405 | 0 |  |  | 0 |  |  |  | 0 |
| Kakamega | 1124 | 103 | 20 | 1247 | 9 |  |  | 9 |  |  |  | 0 |
| Kericho | 665 | 220 | 160 | 1045 | 5 |  |  | 5 |  |  |  | 0 |
| Kiambu | 1144 | 188 | 876 | 2208 | 11 |  | 5 | 16 |  |  | 4 | 4 |
| Kilifi | 284 | 270 | 137 | 691 | 0 |  |  | 0 |  |  |  | 0 |
| Kirinyaga | 402 | 54 | 147 | 603 | 4 |  |  | 4 |  |  |  | 0 |
| Kisii | 726 | 791 | 411 | 1928 | 9 | 5 |  | 14 |  | 5 |  | 5 |
| Kisumu | 1018 | 343 | 428 | 1789 | 11 | 12 |  | 23 |  | 11 |  | 11 |
| Kitui | 522 | 33 | 202 | 757 | 0 |  |  | 0 |  |  |  | 0 |
| Kwale | 102 | 54 | 101 | 257 | 6 |  |  | 6 |  |  |  | 0 |
| Laikipia | 276 | 92 | 48 | 416 | 0 |  |  | 0 |  |  |  | 0 |
| Lamu | 22 | 20 | 68 | 110 | 0 |  |  | 0 |  |  |  | 0 |
| Machakos | 801 | 191 | 125 | 1117 | 5 |  |  | 5 |  |  |  | 0 |
| Makueni | 470 | 20 | 46 | 536 | 4 |  |  | 4 |  |  |  | 0 |
| Mandera | 246 | 40 | 48 | 334 | 0 |  |  | 0 |  |  |  | 0 |
| Marsabit | 218 | 21 | 175 | 414 | 0 |  |  | 0 |  |  |  | 0 |
| Meru | 596 | 98 | 912 | 1606 |  |  | 2 | 2 |  |  | 2 | 2 |
| Migori | 216 | 261 | 161 | 638 | 0 |  |  | 0 |  |  |  | 0 |
| Mombasa | 664 | 433 | 69 | 1166 | 12 | 23 |  | 35 |  | 17 |  | 17 |
| Murang'a | 542 | 186 | 183 | 911 | 0 | 0 | 3 | 3 |  |  |  | 0 |
| Nairobi | 2341 | 2013 | 473 | 4827 | 99 | 137 | 40 | 276 |  | 143 | 24 | 167 |
| Nakuru | 1276 | 276 | 181 | 1733 | 6 | 14 |  | 20 |  | 8 |  | 8 |
| Nandi | 202 | 77 | 35 | 314 | 0 |  |  | 0 |  |  |  | 0 |
| Narok | 133 | 81 | 200 | 414 | 6 |  |  | 6 |  |  |  | 0 |
| Nyamira | 264 | 18 | 87 | 369 | 0 |  |  | 0 |  |  |  | 0 |
| Nyandarua | 118 | 33 | 186 | 337 | 0 |  |  | 0 |  |  |  | 0 |
| Nyeri | 754 | 33 | 430 | 1217 | 6 | 4 | 5 | 15 |  | 3 |  | 3 |
| Samburu | 136 | 42 | 178 | 356 | 0 |  |  | 0 |  |  |  |  |
| Siaya | 460 | 54 | 94 | 608 | 0 |  |  | 0 |  |  |  | 0 |
| Taita Taveta | 236 | 35 | 88 | 359 | 0 |  |  | 0 |  |  |  | 0 |
| Tana River | 98 | 27 | 63 | 188 | 0 |  |  | 0 |  |  |  | 0 |
| Tharaka-Nithi | 108 | 55 | 483 | 646 | 0 |  |  | 0 |  |  |  | 0 |
| Trans Nzoia | 271 | 48 | 33 | 352 | 0 |  |  | 0 |  |  |  | 0 |
| Turkana | 191 | 50 | 117 | 358 | 20 | 36 | 3 | 0 |  | 28 | 3 | 0 |
| Uasin Gishu | 871 | 480 | 57 | 1408 | 0 |  |  | 59 |  |  |  | 31 |
| Vihiga | 162 | 10 | 88 | 260 | 0 |  |  | 0 |  |  |  | 0 |
| Wajir | 88 | 40 | 54 | 182 | 0 |  |  | 0 |  |  |  | 0 |
| West Pokot | 142 | 35 | 127 | 304 |  |  |  | 0 |  |  |  | 0 |

*Source: Kenya Harmonized health facility survey 2019 **Source: Kenya Healthcare Federation Survey 2020
